# Distribution patterns of carbohydrates in murine glycocalyx

**DOI:** 10.1080/13102818.2014.999214

**Published:** 2015-01-29

**Authors:** Velichka Pavlova, Tsvetelina Paunova-Krasteva, Stoyanka Stoitsova, Elena Nikolova

**Affiliations:** ^a^ Department of Experimental Morphology, Institute of Experimental Morphology, Pathology and Anthropology with Museum, Bulgarian Academy of Sciences, Sofia, Bulgaria; ^b^ Department of General Microbiology, The Stefan Angeloff Institute of Microbiology, Bulgarian Academy of Sciences, Sofia, Bulgaria

**Keywords:** carbohydrate moieties, glycocalyx, lectins, microscopy

## Abstract

Enterocytes are unique cells governing an array of processes. They are covered by the gut glycocalyx, which is an extraneous carbohydrate-rich coat and an integral part of the plasma membrane. The intestinal glycocalyx and secreted mucins constitute a glycosylated milieu which has a number of physiological and protective functions. One of the important functions of the glycocalyx is its barrier function against microbial adherence to different membrane glycolipids. Thus, the glycocalyx is an important part of the mucosal immune system in newborns. The aim of our study was to identify the carbohydrates in the small bowel glycocalyx of Balb/c mice at different ages. We used plant lectins with different sugar specificities. Fluorescein-labelled lectins binding different carbohydrate moieties were detected using confocal laser scanning microscopy. Biotinilated lectins were used for transmission electron microscopy observations of the constituents of the gut glycocalyx at different periods of postnatal development in mice. Different carbohydrate moieties that were identified in the murine intestinal glycocalyx followed different distribution patterns and characteristics. Carbohydrates present on the mucus surface depended on tissue localization, cell type and stage of development. The distribution and mucins glycosylation could be of interest in analysing the response of the mucosal barrier to intestinal pathogens causing infection or inflammation.

## Introduction

Enterocytes are unique cells governing an array of processes. They are covered by a specific coat of carbohydrates responsible for gut colonization – the gut glycocalyx. It plays an important role as a barrier against the attachment of different micro-organisms like bacteria, viruses and protozoa. Thus, the glycocalyx is an integral part of the mucosal immune system in newborns. The distribution of carbohydrates on the luminal surface along the intestine not only depends on the part of the organ, but is also age related.[1]

Plant lectins (often named as agglutinins) can bind reversibly to specific monosaccharaides or oligosaccharides. It is considered that the biological function of lectins is to provide defence against different kinds of plant-eating organisms. They may also serve as a depot of nitrogen or as specific recognition factors. At the same time, lectins may interact with glycoconjugates of other organisms.[2] Many studies have been conducted to trace the biological effect of agglutinins on living organisms. For example, there are reports showing that a number of plant lectins are stable in the rodent gut and interact with the mucosal epithelium after animal feeding.[3] Moreover, some plant lectins can be translocated across the gut in humans and in mice,[4] suggesting that lectins could serve as effective mucosal immunogens. There is growing interest in the use of lectins as mucosal vaccine targeting agents, allowing orally administered drugs and vaccines to be more efficient.[5] The gastrointestinal tract could be favoured for mucosal delivery, because certain intestinal cells could offer a portal for absorption of colloidal delivery vehicles in relation to their specific carbohydrate covering.[6] For instance, enterocyte-specific targeting by WGA increases the transepithelial transport, which is of interest for drug development. Another interesting group of intestinal cells, M cells, seem important for the uptake of enteropathogenic micro-organisms and reoviruses. The precise carbohydrate covering of human intestinal M cells is, however, still under investigation.[7,8]

Wheat germ agglutinin (WGA) is a nontoxic lectin which is derived from wheat flour and is highly resistant against gastric digestion. WGA binds specifically to *N*-acetyl-D-glucosamine of epithelial cells and M cells.[9] It has been shown that WGA can stimulate the secretion of liquor and increase alpha-amylase activity in rats.[10] At the same time, *N*-acetyl-D-glucosamine serves as a building block for many oligosaccharides present in maternal milk. It can also facilitate the colonization of the gut by *Bifidobacterium* species in infants. The glycoconjugates composition of intestinal epithelial cells depends not only on the gut microbial flora, but also on its interaction with dietary constituents altering the mucosal architecture and the activity of endocrine cells.[11,12]

Concanavalin A (Con A) is a lectin from Jack bean. It binds specifically glycoproteins and glycolipids containing *α*-d-mannosyl and *α*-d-glucosyl residues. It can interact both with intestinal epithelial cells and with macrophages, stimulating their production of NO and proinflammatory cytokines.[13]

The aim of the present study was to trace age-dependent changes in carbohydrates in the small bowel glycocalyx of Balb/c mice.

## Materials and methods

Balb/c murine intestinal explants from animals of different ages, starting from days 5, 10, 20 and 30, were used. To prepare samples for transmission electron microscopy (Opton EM 109), intestinal explants were blocked with bovine serum albumin (BSA), using a standard pre-embedding protocol, and incubated with biotinilated WGA (25 μg/mL, Vector Laboratories Inc.) or with biotinilated Con A (25 μg/mL, Vector Laboratories Inc) overnight. Then, streptavidin colloidal gold solution (10 nm particles, Sigma) was added overnight. Small sections of duodenum, jejunum or ileum were embedded in resin (Durcopan) and processed for ultra-thin sectioning. After contrasting, grids were observed under transmission electron microscope at different magnifications. Fluorescein-labelled lectins binding different carbohydrate moieties were detected using fluorescent microscopy or confocal laser scanning microscopy (CLSM). We performed cryo-sectioning of different gut parts from animals at all ages. Ultra-thin cryo sections were fixed in 4% formaldehyde solution, blocked with BSA and then stained with fluorescein-labelled WGA (25 μg/mL) or with fluorescein-labelled Con A (25 μg/mL) overnight at 4 °C.[14] After washing with 1.5% BSA for 10 min, sections were treated with ethidium bromide solution (2.4:500 in phosphate buffered saline, 0.25 mL volume was used for each tissue sample) for staining of the nuclei and observed under a Nokon Eclipse Ti-E confocal microscope or an Opton fluorescence microscope equipped with an HBO-200 source of excitation light.

All animal procedures were carried out in accordance with the guidelines of the Animal Ethics Committee at IEMPAM-BAS.

## Results and discussion

The differences in the distribution patterns of sugar residues in the gut lumen could be one explanation for the site-specificity of certain pathogens.[1] Glycoconjugates covering the mucosal surface of intestinal epithelial cells could act as attachment sites for intestinal microbiota. At the same time, some specific bacteria could control lipopolysaccharide concentrations regulating the outcome of inflammatory and metabolic diseases.[15,16] The hydrolysis of carbohydrates by bacteria favours the host's digestion but certain types of microbes can induce carbohydrate secretion by the host.[17] The type of nutrient intake, along with the stage of postnatal development, is an important factor for the distribution of carbohydrates in the intestines of developing animals. These differences could be important determinants in cell–cell and cell–matrix interactions.

**Figure 1.**
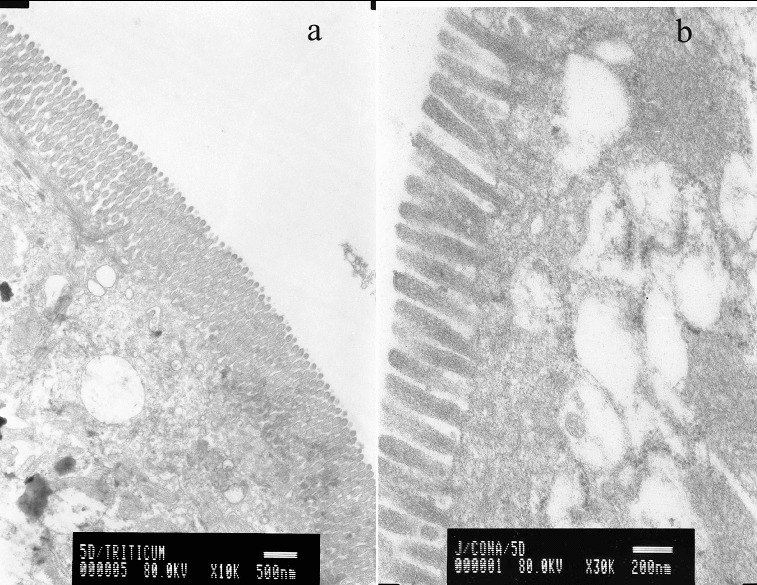
Electron microscopy micrographs of murine gut explants at day 10 postpartum treated with WGA (a) or with Con A (b).

WGA specifically binds N-acetyl-β-glucosamine oligomers. We detected that colloidal-gold staining of WGA was abundant on the surface of whole villi covering the duodenum, jejunum and ileum of murine small intestine (Figure 1(a)). Age-related expression patterns of the latter carbohydrates were observed. Con A, which specifically binds mannose, was evenly distributed on the tip of microvilli, as detected by transmission electron microscopy (Figure 1(b)).

**Figure 2.**
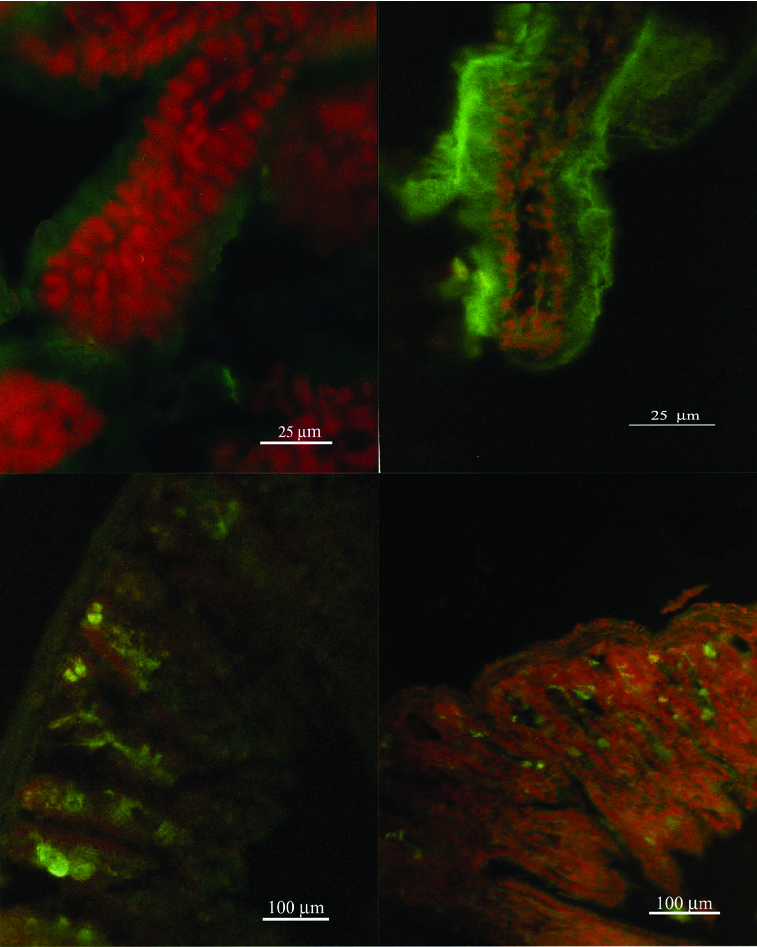
Fluorescent microscopy of gut explants treated with WGA at day 5 to day 30 postpartum. Maximum activity was detected at day 10.

**Figure 3.**
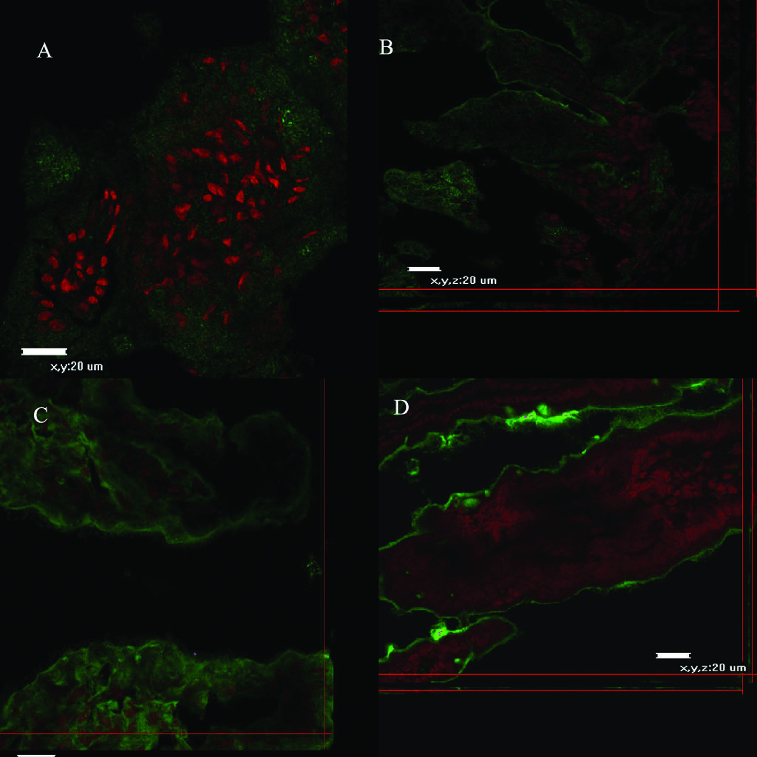
Confocal micrographs of small intestinal explants treated with WGA (a–d, days 5–30).

**Figure 4.**
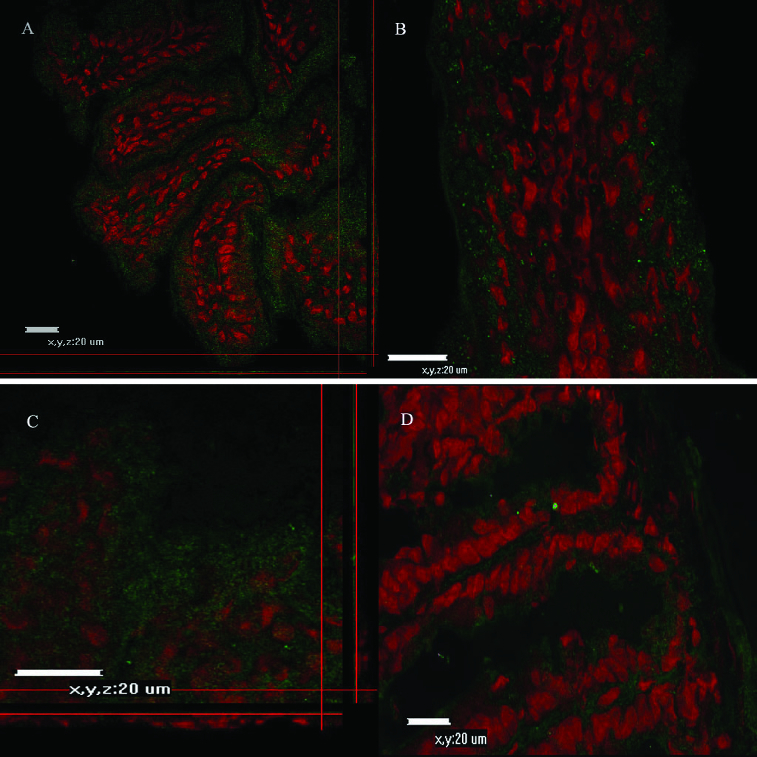
Confocal micrographs of small intestinal explants treated with Con A at different days of development.

CLSM and fluorescent microscopy (Figure 2) observations were performed to trace the binding of fluorescein-labelled Con A or WGA lectins. Confocal microscope micrographs (Figures 3 and 4) revealed age-dependent distribution patterns of carbohydrate moieties in the murine intestinal glycocalyx of developing animals. Con A was more abundant at days 5–10 (Figure 4), whereas at day 20 and day 30 there was less expression of fluorescent dye on the tip of intestinal villi. There was noticeable expression also at the gut wall of the small intestinal explants. At the same time, Con A expression was more obvious in the duodenum explants and less obvious in the jejunum and ileum explants. WGA expression showed similar distribution at all examined days and for the three parts of the murine gut (Figures 3 and 4).

Taken together, these results show that the reaction of mannose with Con A was detected from days 10 on the surface of murine enterocytes, whereas N-acetyl-β-glucosamine was constantly present during all days of the examination in all parts of the murine gut. As a next step in our studies, the distribution patterns of fucose, galactose and sialic acid on murine enterocytes are currently under examination.

## Conclusions

The present study showed that there are differences in lectins binding during the first 30 days of murine life, suggesting a possibility for various micro-organisms to colonize the developing gut. Different carbohydrate moieties were identified in the glycocalyx of murine small intestine. They followed different distribution patterns and characteristics. Since the carbohydrates present on the mucous surface depend on the tissue localization, cell type and the stage of development, the distribution and mucins glycosylation could be of interest in analysing the response of the mucosal barrier to intestinal pathogens causing infection or inflammation. 

## Disclosure statement

No potential conflict of interest was reported by the authors.
